# Merging Generative Linguistics and Psycholinguistics

**DOI:** 10.3389/fpsyg.2018.02283

**Published:** 2018-11-28

**Authors:** Jordi Martorell

**Affiliations:** Basque Center on Cognition Brain and Language, Donostia-San Sebastián, Spain

**Keywords:** merge, syntax, sentence processing, prediction, oscillations, eye tracking

## Introduction

There have been constant debates about the connection between the theoretical postulates of generative linguistics (Chomsky, 1965, 1995) and the experimental research carried out in psycho-/neurolinguistics (see Poeppel and Embick, 2005; Embick and Poeppel, 2015). This cross-disciplinary relationship has been approached from noticeable distinct positions, including some views taking generative accounts as well-suited on their own for experimental investigation (e.g., Marantz, 2005; Sprouse and Hornstein, 2016), others proposing a reconsideration of certain generative assumptions about processing issues (e.g., Lewis and Phillips, 2015; Mancini, 2018), and others openly advocating for adopting alternative linguistic frameworks (e.g., Townsend and Bever, 2001; Ferreira, 2005; Jackendoff, 2007; Christiansen and Chater, 2017). Although real-time processing data are generally consistent with theoretical considerations (see Phillips and Wagers, 2007; Lewis and Phillips, 2015), it is noteworthy that experimental results from psycholinguistics are rarely incorporated into generative accounts (cf. Chomsky, 2005). This limited interaction between disciplines is motivated by the competence-performance distinction (Chomsky, 1965), also instantiated as the computational and algorithmic levels of analysis (Marr, 1982), with linguistics developing formal accounts of grammatical phenomena (i.e., competence/computation) independently from the psycholinguistic evidence about how they manifest in real-time processing (i.e., performance/algorithm).

One way to facilitate the cross-talk between linguistics and psycholinguistics is to assume a transparent computation-to-algorithm mapping in which these two levels represent distinct timescales of the *same* cognitive system: the computation denotes its offline properties and the algorithm its online execution (Lewis and Phillips, 2015). As a result, the algorithm might involve the processing-sensitive internal stages of the computation (Lewis and Phillips, 2015; see also Mancini, 2018). In this view, the two disciplines could directly inform each other to establish a unified computational-algorithmic account of language—a *(psycho)linguistic theory* (Embick and Poeppel, 2015)—where their respective insights provide mutual constraints for explaining fundamental aspects of the linguistic system. Accounts along these lines have recently been proposed to capture phenomena such as grammaticality illusions (see Lewis and Phillips, 2015) and agreement (Mancini, 2018). This opinion article aims to show how this cross-disciplinary approach can be effectively extended to a new area: the generation of syntactic structures.

## Building syntactic structures in real time

A critical requirement for advancing the collaboration between generative linguistics and psycholinguistics is explaining how sentential representations are incrementally constructed in real time (i.e., roughly on a word-by-word basis). While the surface form of sentences comprises words ordered linearly, the sentence-level meaning is established by the *abstract* hierarchical structure arising from certain syntactic relations (see Everaert et al., 2015). Given that syntactic structure is not obvious from the external input, it must be generated *internally*, presumably in accordance with the rules and constraints of some sort of real-time structure-building mechanism. In this respect, any computational-algorithmic account of structure generation would require specifying the formal properties of such a mechanism and, in addition, determining how its time-dependent steps are executed during online sentence processing^1^.

In generative linguistics, this structure-building mechanism is formally instantiated by the computational operation *Merge* (Chomsky, 1995), which recursively selects and combines syntactic units to create new structures that are hierarchically organized (see also Chomsky, 2013)^2^. Importantly, Merge is strictly conceived at the competence domain as an offline computation without real-time implications. This is evidenced by the fact that Merge generates sentences from right to left, namely in the opposite direction to that required by language processing, and consequently remains uncommitted on how words are hierarchically combined in real time (see Chesi, 2012, 2015 for a detailed discussion). Phillips (1996) precisely resolves such timing concerns by proposing that Merge proceeds in reverse order (i.e., from left to right) during processing, thus providing a better characterization of Merge in computational-algorithmic terms (see also Phillips, 2003).

Interestingly, recent neurolinguistic research has shown that neural oscillations exhibit subtle sensitivity to abstract syntactic structure, either tracking the hierarchical levels of speech sequences without prosodic cues (Ding et al., 2016) or showing spectro-temporal modulations driven by underlying hierarchical structure (Nelson et al., 2017). This reflection of hierarchical structure in oscillatory patterns suggests that the structure-building mechanism could incrementally group words together in a Merge-like manner during sentence comprehension (cf. Frank and Christiansen, 2018). Note that both Nelson et al.'s and Ding et al.'s results seem to index the *bottom-up* integration of sensory information *after* its presentation (i.e., post-stimulus evoked activity, see Ding and Simon, 2014; Ding et al., 2017). Likewise, Merge is currently formalized as initially selecting and subsequently combining syntactic units (see Chomsky, 2013; Murphy, 2015) and, in processing terms, this would imply the integration (i.e., selection and combination) of syntactic information that has already occurred as external input. Thus, in line with the proposal that Merge could be operationalized in real time (Phillips, 1996, 2003), the reported oscillatory patterns might reflect the output of a Merge-like mechanism that integrates chunks of bottom-up information quickly and in a hierarchically-structured way.

However, sentence processing is not limited to bottom-up integrative analyses. Indeed, recent proposals and findings have underscored the role of *top-down* predictive processes (Altmann and Mirković, 2009; DeLong et al., 2014; Staub, 2015; Kuperberg and Jaeger, 2016; cf. Huettig and Mani, 2016). In other words, besides the incremental integration of bottom-up information from the sensory input, sentence processing also involves top-down expectations regarding upcoming information *before* its actual occurrence, which may subsequently manifest as facilitated integration of the predicted material. This raises the question of whether the incremental Merge-like mechanism that generates syntactic structures also incorporates a predictive component, with top-down syntactic predictions preceding and also modulating the bottom-up processing of sentences.

## A predictive structure-building mechanism?

As noted, the strictly integrative formalization of Merge limits its suitability for properly capturing how syntactic structures could be built predictively. Thus, computational-algorithmic adequacy requires that the Merge-like mechanism involve two computational substeps: a top-down stage for predicting syntactic structure and a bottom-up stage for syntactically integrating actual input (cf. Chesi, 2012, 2015 for an alternative predictive account). Note that the latter (but not the former) stage is reflected in the left-to-right Merge proposed by Phillips (1996, 2003). Moreover, regarding which specific type of syntactic information is preactivated, one possibility is that syntactic prediction takes place probabilistically for a broad range of syntactic units (Levy, 2008). However, since sentences are potentially infinite, a more parsimonious strategy might be to avoid the prediction of optional syntactic information (i.e., adjuncts), similarly to the comprehension model proposed by Hale (2011). Therefore, a plausible hypothesis could be that, under normal circumstances, top-down syntactic expectations mainly concern the core structural elements required in sentences, namely verbs and their arguments (e.g., subjects and objects), while optional information such as adjuncts (e.g., adverbs) primarily involves bottom-up processing without prior activation. For instance, in the sentence “children ate cookies yesterday,” the processing of the subject “children” would preactivate the syntactic position for the sentential-level mandatory verb (e.g., “ate”). Since the processed verb is transitive, it would trigger the prediction of its syntactically-required object (e.g., “cookies”) but not of any optional adverb (e.g., “yesterday”)^3^.

Crucially, this hypothesis is consistent with behavioral evidence (mostly) from eye-tracking experiments. First, in verb-final languages such as Japanese or German, syntactic violations associated with arguments show reading disruptions before the verb appears, indicating that some type of verb-related structural information is preactivated (see Phillips and Lau, 2004). In addition, the presence of arguments (relative to adjuncts) results in facilitated integration of clause-final verbs (Levy and Keller, 2013), which suggests that mandatory information strongly contributes to the prediction of syntactic units such as verbs (although this facilitation might be lexical in nature, see Husain et al., 2014). Second, using the visual-world paradigm, anticipatory eye-movements toward images referring to object nouns are selectively triggered in specific syntactic contexts (Kamide et al., 2003), and are modulated by lexico-syntactic factors such as verb (in)transitivity (Arai and Keller, 2013), thereby providing compelling evidence for the prior activation of these obligatory syntactic units under particular structural conditions. Furthermore, syntactic-category violations (such as subject/object nouns instead of verbs and vice versa) presented in the parafovea seem to reduce skipping rates (Brothers and Traxler, 2016; Snell et al., 2017), suggesting that syntactic contexts generate expectations for upcoming words with such syntactic specifications before they are fixated.

Interestingly, previous reading findings could be reinterpreted in terms of the current hypothesis that syntactic prediction is largely confined to verbs and their syntactically-required elements. Specifically, Staub (2007) found that a postverbal noun like “the vet” produced an early reading disruption when it could be the subject of the upcoming main clause, i.e., after an intransitive verb (“When the dog arrived the vet …”), relative to being the object of the embedded clause, i.e., after a transitive verb (“When the dog scratched the vet …”). While Staub (2007) attributed this reading slowdown to a general processing cost for a noun starting a main clause, such effects could reflect the combination of two predictive aspects: (i) a processing cost for an unexpected postverbal noun in an intransitive context (and, in turn, a processing advantage for the predicted object noun following a transitive verb), and (ii) increased processing demands for a subject generating the prediction for the upcoming main-clause verb^4^. Moreover, a predictive explanation along these lines also seems to account for the observation that arguments lead to faster reading times compared to adjuncts (see Tutunjian and Boland, 2008), reflecting facilitated integration for expected arguments. Therefore, converging evidence from psycholinguistic experiments suggests that the Merge-like mechanism should indeed include a predictive component that selectively generates expectations for structurally-required elements.

## Conclusion

In summary, previous behavioral evidence seems to support the possibility that syntactic prediction is a significant property of the Merge-like structure-building mechanism and, more concretely, the hypothesis that such expectations are primarily generated for core syntactic units (i.e., verbs and their arguments). However, more research is needed to narrow down the computational-algorithmic explanation of predictive structure generation, which should be extendable to a broader variety of structural contexts (see Gibson, 2006; Staub and Clifton, 2006; Linzen and Jaeger, 2015; Omaki et al., 2015) and typologically different languages (e.g., head-final). For example, do top-down predictions underlie the projection of single syntactic nodes (as shown in the example sentence above) or the retrieval of structural templates (as in alternative proposals, e.g., Townsend and Bever, 2001; Jackendoff, 2007; Bever and Poeppel, 2010)? Also, what is the representational format of the predicted content: high-level abstract properties of syntactic types like “verb” (as assumed here) and/or low-level sensory (e.g., orthographic) forms associated with syntactic categories (e.g., Dikker et al., 2010; Farmer et al., 2015)? Moreover, regarding neural implementation, the details of a predictive structure-building mechanism should be explicitly linked to the oscillatory dynamics of language comprehension and production (Lewis and Bastiaansen, 2015; Molinaro et al., 2016; Meyer, 2017; Segaert et al., 2018). In line with the *(psycho)linguistic theory* explored here, reciprocal contributions from theoretical and experimental (both behavioral and neurophysiological) work are fundamental to address such issues and shed further light on the multilevel nature of syntactic structures.

In conclusion, the above argumentation about syntactic structure generation shows that, assuming a transparent mapping between computation and algorithm, psycholinguistic findings on predictive processing can be effectively combined with formalizations of computational operations such as Merge from generative linguistics to refine our understanding of the structure-building mechanism. Therefore, such a cross-disciplinary approach supposes a promising strategy toward a comprehensive multilevel theory of language integrated within cognitive neuroscience.

## Author contributions

The author confirms being the sole contributor of this work and has approved it for publication.

### Conflict of interest statement

The author declares that the research was conducted in the absence of any commercial or financial relationships that could be construed as a potential conflict of interest. The reviewer CC and handling Editor declared their shared affiliation.

## Footnotes

^1^This article focuses on sentence comprehension, but the same concerns apply to language production (see Chesi, 2012; Momma and Phillips, 2018).

^2^Other frameworks hypothesize alternative operations with different formal characteristics and processing implications (e.g., Unification, Jackendoff, 2007). Due to space limitations, such alternatives cannot be explored here (see Phillips and Lau, 2004, for discussion).

^3^As a reviewer points out, this distinction between arguments and adjuncts could be confounded by co-occurrence factors. Indeed, empirical research (e.g., corpora-based probabilistic measures, see Hale, 2016) will be needed to assess such confounds.

^4^These two effects would correspond to surprisal (Levy, 2008) and entropy reduction (Hale, 2003, 2011), respectively, under corpora-based probabilistic accounts of syntax (see Hale, 2016).
